# Supplementary material for: Explainable deep learning for disease activity prediction in chronic inflammatory joint diseases
Source: PLOS Digit Health. 2024 Jun 27;3(6):e0000422. doi: 10.1371/journal.pdig.0000422 (PMC11210792; doi:10.1371/journal.pdig.0000422)
Supplement: S1 Table — (PDF) [file pdig.0000422.s001.pdf]

Dataframe	demographics	clinical measures	medications	PROM
Features	date_of_birth,<br>gender,<br>date_first_symptoms,<br>date_diagnosis	date,<br>weight_kg,<br>das283bsr_score,<br>asdas_score,<br>n_swollen_joints,<br>n_painfull_joints,<br>bsr,<br>n_painfull_joints_28,<br>height_cm,<br>crp,<br>hb,<br>n_enthesides,<br>mda_score,<br>joints_type,<br>anti_ccp,<br>ra_crit_rheumatoid_factor,<br>smoker,<br>haq_score	medication_generic_drug,<br>medication_drug_classification,<br>medication_dose,<br>date,<br>is_start	date,<br>pain_level_today_RADAI,<br>morning_stiffness_duration_RADAI,<br>activity_of_rheumatic_disease_today_RADAI
