# Supplementary material for: Explainable deep learning for disease activity prediction in chronic inflammatory joint diseases
Source: PLOS Digit Health. 2024 Jun 27;3(6):e0000422. doi: 10.1371/journal.pdig.0000422 (PMC11210792; doi:10.1371/journal.pdig.0000422)
Supplement: S2 Table — (PDF) [file pdig.0000422.s002.pdf]

	mean	std	missing (%)
weight_kg	72.78	16.03	30.21
das283bsr_score	3.02	1.40	14.45
asdas_score	2.34	1.03	84.63
n_swollen_joints	2.54	4.18	4.94
n_painfull_joints	3.42	5.51	18.70
bsr	16.33	15.91	0.98
n_painfull_joints_28	2.99	4.86	14.36
height_cm	167.06	9.23	34.72
crp	6.56	11.33	26.74
hb	13.80	1.36	36.64
n_enthesides	1.31	2.40	78.62
mda_score	1.68	1.33	0.31
haq_score	0.77	0.71	40.16
