# Supplementary material for: Explainable deep learning for disease activity prediction in chronic inflammatory joint diseases
Source: PLOS Digit Health. 2024 Jun 27;3(6):e0000422. doi: 10.1371/journal.pdig.0000422 (PMC11210792; doi:10.1371/journal.pdig.0000422)
Supplement: S3 Table — (PDF) [file pdig.0000422.s003.pdf]

**Feature**	**Category**	**Percentage (%)**	**Missing (%)**
smoker	i_have_never_smoked	16.18	65.06
	i_am_a_former_smoker_for_more_than_a_year	11.05	
	i_am_currently_smoking	7.71	
anti_ccp	positive	36.34	41.26
	negative	22.40	
ra_crit_rheumatoid_factor	positive	51.44	18.49
	negative	30.07	
joints_type	28.0	65.36	0.05
	44.0	22.39	
	68.0	12.20	
