# Supplementary material for: Explainable deep learning for disease activity prediction in chronic inflammatory joint diseases
Source: PLOS Digit Health. 2024 Jun 27;3(6):e0000422. doi: 10.1371/journal.pdig.0000422 (PMC11210792; doi:10.1371/journal.pdig.0000422)
Supplement: S4 Table — (PDF) [file pdig.0000422.s004.pdf]

	mean	std	missing (%)
medication_dose	253.32	459.76	0.25
