# Supplementary material for: Explainable deep learning for disease activity prediction in chronic inflammatory joint diseases
Source: PLOS Digit Health. 2024 Jun 27;3(6):e0000422. doi: 10.1371/journal.pdig.0000422 (PMC11210792; doi:10.1371/journal.pdig.0000422)
Supplement: S5 Table — (PDF) [file pdig.0000422.s005.pdf]

Feature	Category	Percentage (%)	Missing (%)
medication_generic_drug	methotrexate	23.34	0.00
	prednisone	17.32	
	rituximab	10.26	
	adalimumab	8.09	
	etanercept	6.68	
	infliximab	5.77	
	leflunomide	5.70	
	sulfasalazine	5.09	
	Other	3.53	
	golimumab	3.40	
	hydroxychloroquine	2.95	
	tocilizumab	2.69	
	abatacept	2.15	
	certolizumab	2.15	
	secukinumab	0.89	
medication_drug_classification	bDMARD	42.68	0.35
	csDMARD	37.40	
	steroid	17.35	
	tsDMARD	2.23	
is_start	yes	54.96	0.00
	no	45.04	
