# Supplementary material for: Explainable deep learning for disease activity prediction in chronic inflammatory joint diseases
Source: PLOS Digit Health. 2024 Jun 27;3(6):e0000422. doi: 10.1371/journal.pdig.0000422 (PMC11210792; doi:10.1371/journal.pdig.0000422)
Supplement: S6 Table — (PDF) [file pdig.0000422.s006.pdf]

	mean	std	missing (%)
pain_level_today_RADAI	3.01	2.59	0.57
activity_of_rheumatic_disease_today_RADAI	3.14	2.57	0.75
