# Supplementary material for: Explainable deep learning for disease activity prediction in chronic inflammatory joint diseases
Source: PLOS Digit Health. 2024 Jun 27;3(6):e0000422. doi: 10.1371/journal.pdig.0000422 (PMC11210792; doi:10.1371/journal.pdig.0000422)
Supplement: S7 Table — (PDF) [file pdig.0000422.s007.pdf]

Feature	Category	Percentage (%)
morning_stiffness_duration_RADAI	no_morning_stiffness	45.86
	less_than_30_minutes	23.87
	30_minutes_to_1_hour	14.36
	1_to_2_hours	7.10
	2_to_4_hours	3.78
	all_day	2.31
	more_than_4_hours	1.83
**Missing (%)**	0.89	
