# Supplementary material for: Explainable deep learning for disease activity prediction in chronic inflammatory joint diseases
Source: PLOS Digit Health. 2024 Jun 27;3(6):e0000422. doi: 10.1371/journal.pdig.0000422 (PMC11210792; doi:10.1371/journal.pdig.0000422)
Supplement: S8 Table — (PDF) [file pdig.0000422.s008.pdf]

	mean	std	missing (%)
age	65.41	15.71	0.00
age_at_first_symptoms	41.88	15.77	2.52
age_at_diagnosis	44.56	15.06	1.98
