# Supplementary material for: Explainable deep learning for disease activity prediction in chronic inflammatory joint diseases
Source: PLOS Digit Health. 2024 Jun 27;3(6):e0000422. doi: 10.1371/journal.pdig.0000422 (PMC11210792; doi:10.1371/journal.pdig.0000422)
Supplement: S9 Table — (PDF) [file pdig.0000422.s009.pdf]

**Feature**	**Category**	**Percentage (%)**
gender	female	65.17
	male	34.83
**Missing (%)**		0.0
