# Supplementary figures and images for: Explainable deep learning for disease activity prediction in chronic inflammatory joint diseases
Source: PLOS Digit Health. 2024 Jun 27;3(6):e0000422. doi: 10.1371/journal.pdig.0000422 (PMC11210792; doi:10.1371/journal.pdig.0000422)

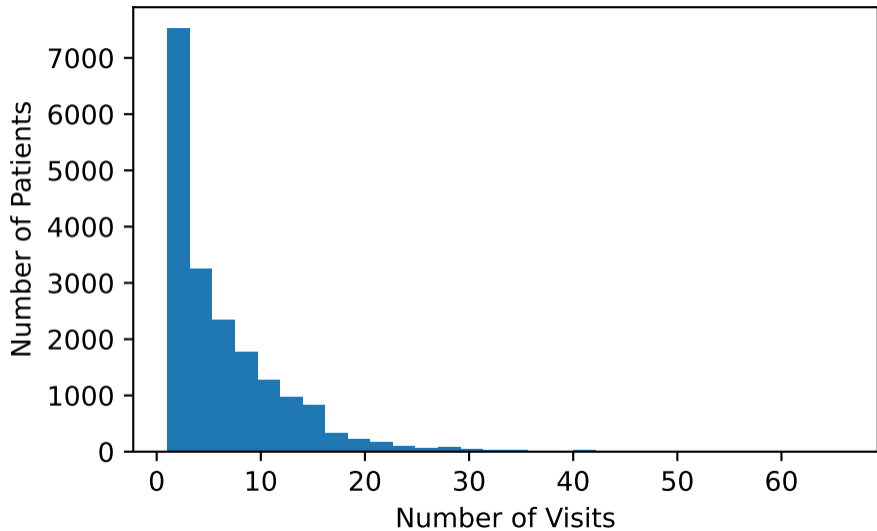

Supplement: S1 Fig — (PDF) [file pdig.0000422.s010.pdf]

DAS28 by Number of Visits

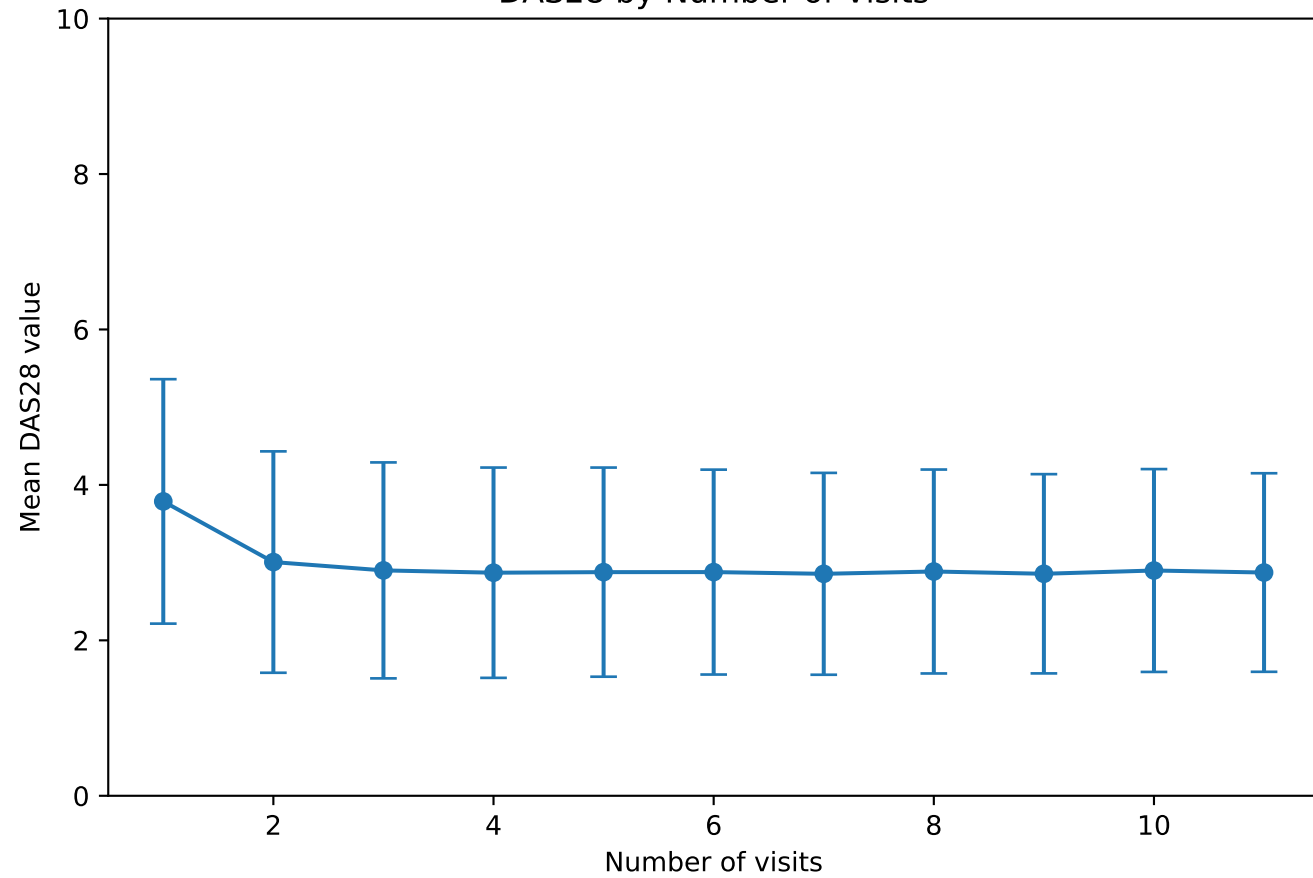

ASDAS by Number of Visits

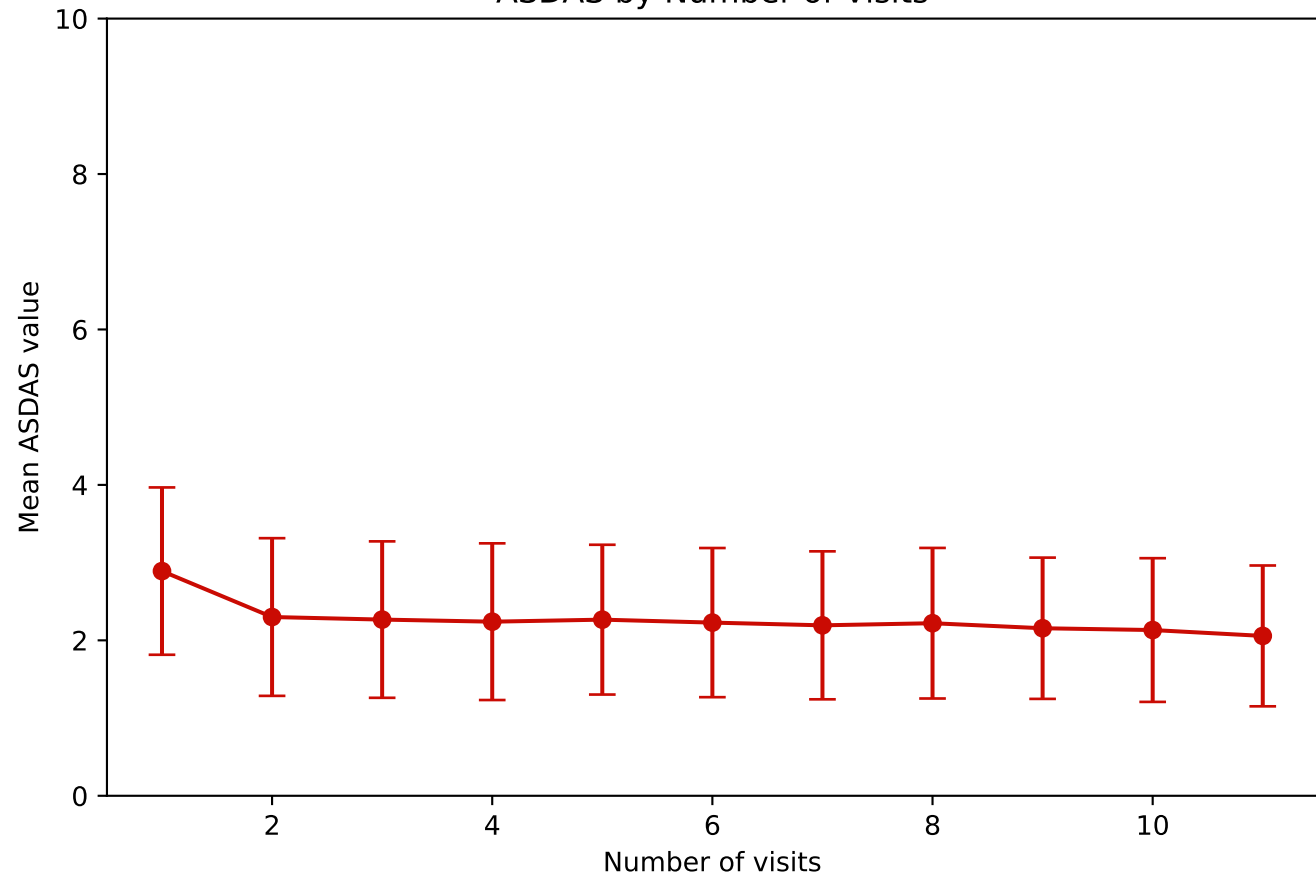

Supplement: S4 Fig — (PDF) [file pdig.0000422.s013.pdf]

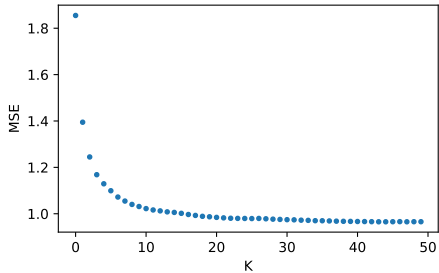

(a) **Smaller  $k$**

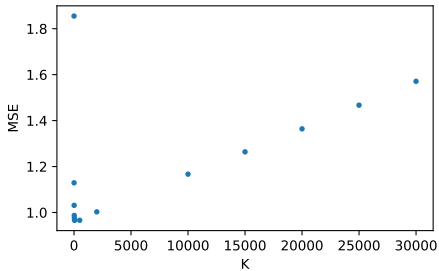

(b) **Larger  $k$**

Supplement: S5 Fig — (PDF) [file pdig.0000422.s014.pdf]

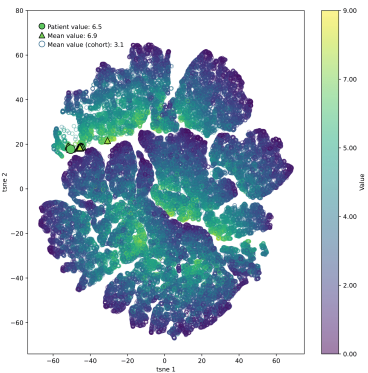

(a) DAS28 value

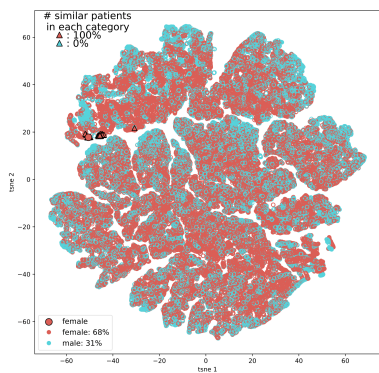

(b) Gender

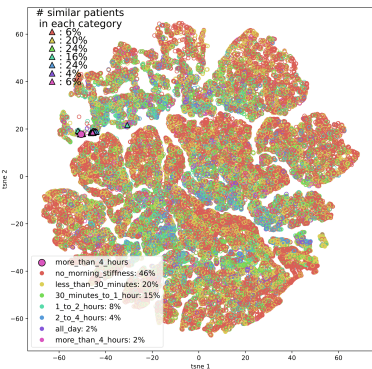

(c) Morning stiffness duration

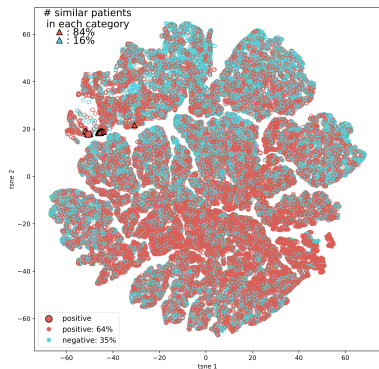

(d) Rheumatoid factor

Supplement: S6 Fig — The DAS28 score and gender show a high level of consistency among the closest neighbors of this patient. Duration of morning stiffness and rheumatoid factors also show slight distribution shifts within the subsets of nearest neighbours. (PDF) [file pdig.0000422.s015.pdf]

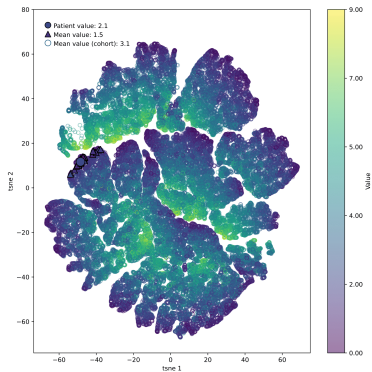

(a) DAS28 value

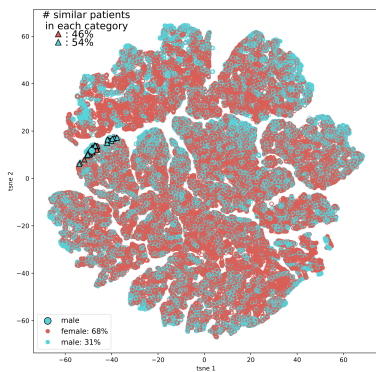

(b) Gender

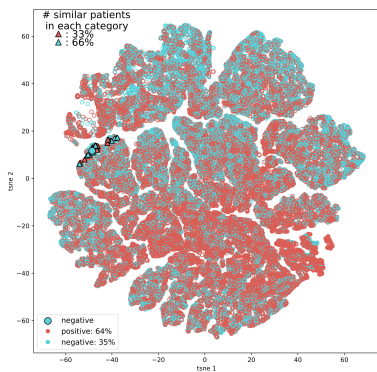

(c) Rheumatoid factor

Supplement: S7 Fig — The patient’s rheumatoid factor status and their low DAS28 value can be observed among the nearest neighbours subset as well. (PDF) [file pdig.0000422.s016.pdf]
