# Supplementary material for: Explainable deep learning for disease activity prediction in chronic inflammatory joint diseases
Source: PLOS Digit Health. 2024 Jun 27;3(6):e0000422. doi: 10.1371/journal.pdig.0000422 (PMC11210792; doi:10.1371/journal.pdig.0000422)
Supplement: S2 Fig — (PDF) [file pdig.0000422.s011.pdf]

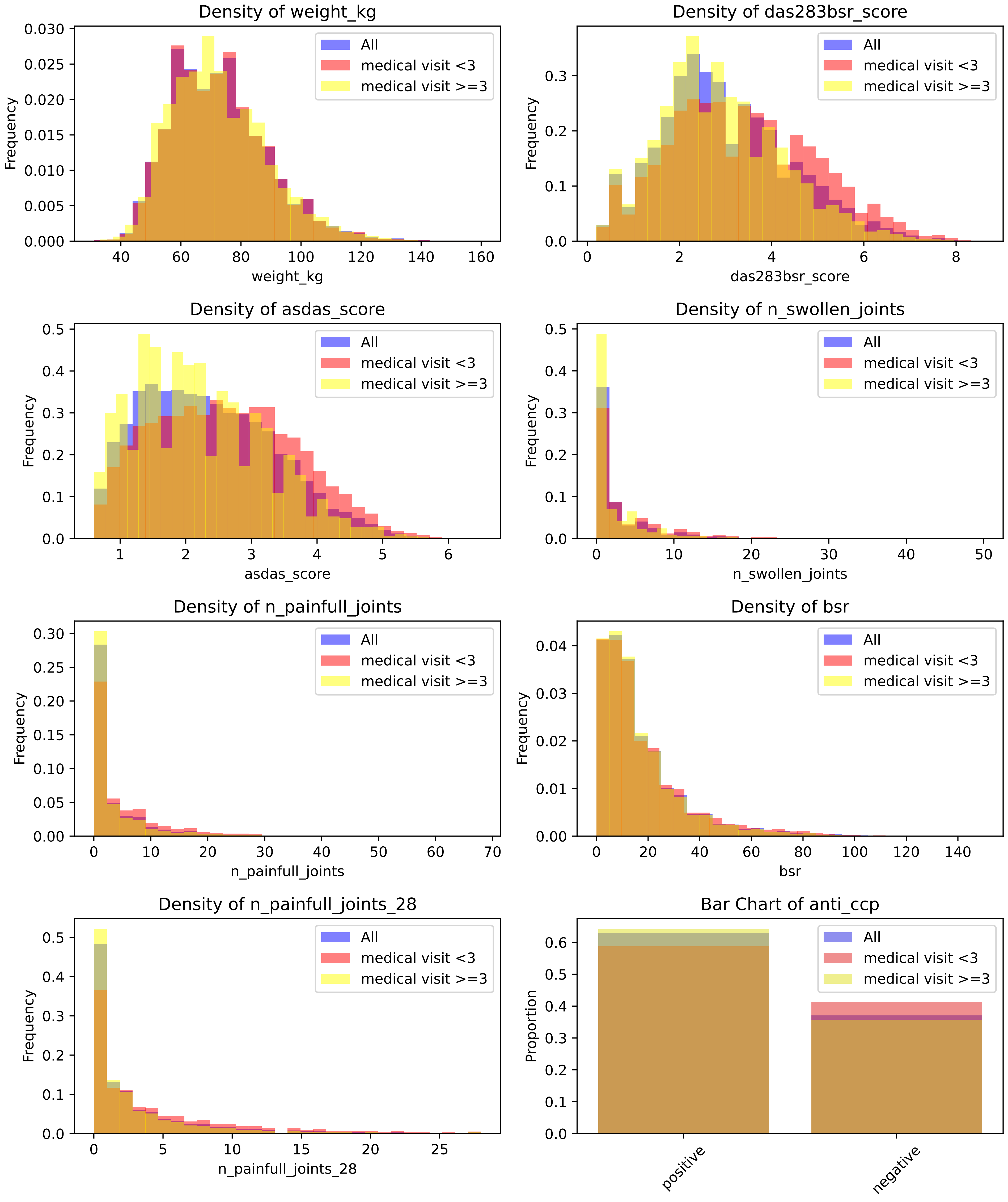
Density of weight_kg
Frequency
weight_kg
Density of das283bsr_score
Frequency
das283bsr_score
Density of asdas_score
Frequency
asdas_score
Density of n_swollen_joints
Frequency
n_swollen_joints
Density of n_painfull_joints
Frequency
n_painfull_joints
Density of bsr
Frequency
bsr
Density of n_painfull_joints_28
Frequency
n_painfull_joints_28
Bar Chart of anti_ccp
Proportion
positive
negative
All
medical visit <3
medical visit >=3
