# Supplementary material for: Explainable deep learning for disease activity prediction in chronic inflammatory joint diseases
Source: PLOS Digit Health. 2024 Jun 27;3(6):e0000422. doi: 10.1371/journal.pdig.0000422 (PMC11210792; doi:10.1371/journal.pdig.0000422)
Supplement: S3 Fig — (PDF) [file pdig.0000422.s012.pdf]

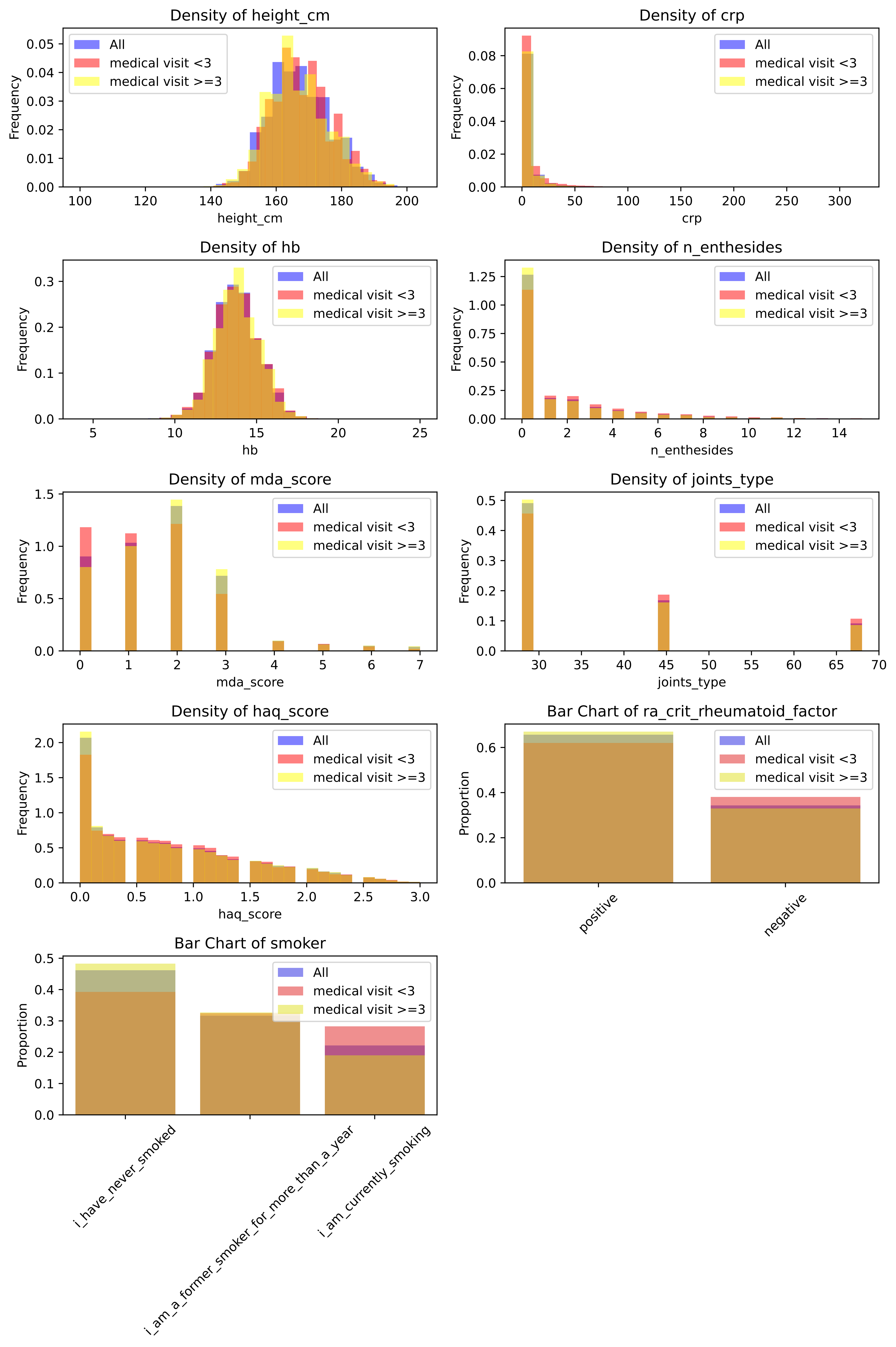
Density of height_cm
Frequency
height_cm
All
medical visit <3
medical visit >=3
Density of crp
Frequency
crp
Density of hb
Frequency
hb
Density of n_enthesides
Frequency
n_enthesides
Density of mda_score
Frequency
mda_score
Density of joints_type
Frequency
joints_type
Density of haq_score
Frequency
haq_score
Bar Chart of ra_crit_rheumatoid_factor
Proportion
positive
negative
Bar Chart of smoker
Proportion
i_have_never_smoked
i_am_a_former_smoker_for_more_than_a_year
i_am_currently_smoking
