# Supplementary material for: Explainable deep learning for disease activity prediction in chronic inflammatory joint diseases
Source: PLOS Digit Health. 2024 Jun 27;3(6):e0000422. doi: 10.1371/journal.pdig.0000422 (PMC11210792; doi:10.1371/journal.pdig.0000422)
Supplement: S1 Text — (PDF) [file pdig.0000422.s017.pdf]

1 S1 Text. Impact of features on prediction

1.1 Error analysis

To evaluate the fairness and robustness of model predictions across subgroups, we computed the correlation between the prediction error and the values of continuous features (Table 1) and the standard deviation of the prediction error across subgroups for categorical features (Table 2). The features showing the highest correlation with model error (e.g. "n_painfull_joints_28") are also closely related to the disease activity scores. For categorical features, only the "smoker" variable shows a significant difference across groups for the ASDAS prediction. The model thus does not exhibit strong biases and performance is relatively robust across various feature subgroups.

Variable	DAS28	ASDAS
age	0.015	-0.041
weight_kg	-0.005	-0.052
crp	0.160	0.367
n_swollen_joints	0.298	0.100
n_painfull_joints	0.326	Not applicable
bsr	0.142	0.171
n_painfull_joints_28	0.351	-0.045
height_cm	-0.006	-0.083
hb	-0.042	-0.097
n_enthesides	0.066	0.061
mda_score	-0.161	-0.088
joints_type	-0.034	-0.006
haq_score	0.145	Not applicable

Table 1: Correlation between continuous features and model error

Variable	DAS28	ASDAS
gender	0.016	0.029
anti_ccp	0.076	0.041
ra_crit_rheumatoid_factor	0.087	0.037
smoker	0.017	0.206

Table 2: Standard deviation of error across groups for categorical features

1.2 Imputation experiments

To additionally evaluate the feature importance results from the patient similarity analysis (Tables 4 and 5), we performed the following experiment. In the test data, we randomly masked out (i.e. imputed as "missing") 10% of three selected features and evaluated the impact on prediction error. We selected one

important feature “n_painfull_joints_28” (AAD 0.41), one of medium importance “mda_score” (AAD 0.56) and one of low importance “weight_kg” (AAD 0.76), and compared the model MSE on the three imputed test datasets. Table 3 shows that imputing important features leads to the highest increase in MSE while imputing a less important feature has almost no impact on the final MSE.

	Original data	n_painfull_joints_28	mda_score	weight_kg
MSE	0.965	1.018	1.000	0.968

Table 3: Imputing features: impact on model performance

1.3 Feature associations experiments

Moreover, we evaluated the systematic biases learned by the model. More specifically, we analyzed the impact on model predictions when artificially modifying the values of the gender and age features. Since these features naturally correlate with disease activity, we wanted to ensure that the model does not systematically learn these correlations. In the raw data, age correlates 0.21 with DAS28, and females have on average a DAS28 of 3.23 versus 2.76 for males.

To analyze the association between gender and DAS28 learned by the model, we artificially changed all the males in the test data to females, and compared the model predictions. Figure 1a shows the difference in predicted DAS28 scores between the males of the test data and the predictions after setting their gender to female. While the model learns that females are associated with higher DAS28 values and predicts higher values for some patients, it does not capture this correlation systematically, the mode of the difference plot being close to 0. The model thus does not learn spurious correlations between gender and disease activity and predicts similar distributions on the original and modified data (Figure 1b).

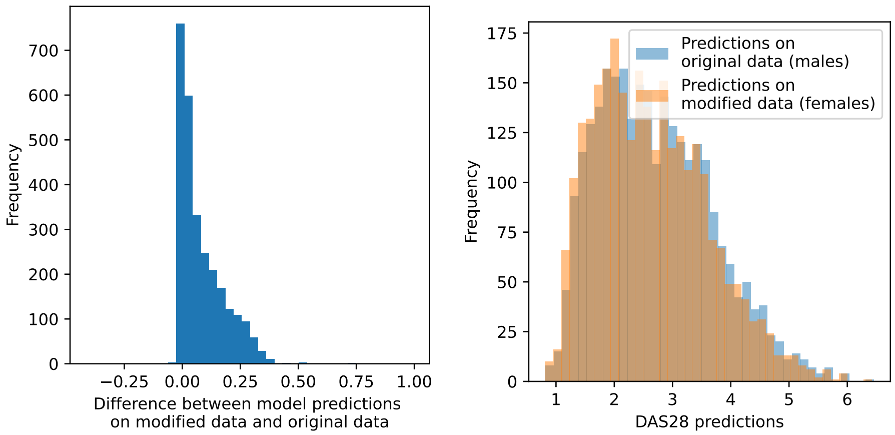

(a) Difference between model predictions on original (male) and modified (female) data

(b) Difference between model predictions on original and modified data

Figure 1: Impact on model predictions when setting all genders to female.

Similarly, to analyse the association between age and predicted disease activity, we set the age to 72 years for all younger patients in the test data. We can again observe that while the difference in predicted scores between the modified data and original data is generally positive, the mode of the difference plot is close to 0 (Figure 2a). The model thus learns that older age is associated with higher disease activity for some patients but not in a systematic way.

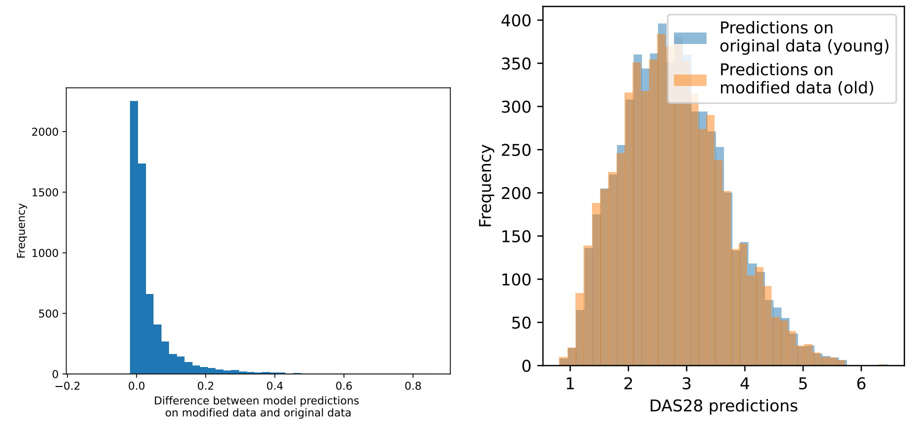

(a) Difference between model predictions on original (young) and modified (old) data

(b) Difference between model predictions on original and modified data

Figure 2: Impact on model predictions when setting the age of all younger patients to 72 years.
