# Supplementary material for: Explainable deep learning for disease activity prediction in chronic inflammatory joint diseases
Source: PLOS Digit Health. 2024 Jun 27;3(6):e0000422. doi: 10.1371/journal.pdig.0000422 (PMC11210792; doi:10.1371/journal.pdig.0000422)
Supplement: Attachment — Submitted filename: scqm_response_to_reviewers.pdf [file pdig.0000422.s018.pdf]

Universität Zürich[UZH]

Zurich, Switzerland

May 13, 2024

RE: Response to Reviewers

Explainable deep learning for disease activity prediction in chronic inflammatory joint diseases

Dear PLOS Digital Health Editorial Team,

We kindly thank the editor and reviewers for their highly constructive feedback and suggestions. We have now included several additional experiments based on the editor and reviewers' suggestions. Please find a point-by-point response below accompanied by a revised manuscript.

We believe that this process has allowed us to greatly strengthen our work.

We would be more than happy to provide additional information if necessary.

Kind regards,

Michael Krauthammer, MD PhD

Department of Quantitative Biomedicine, Chair of Medical Informatics, University of Zurich, Zurich, Switzerland

(on behalf of the authors)

Point by point response: Reviewer 1

We thank the reviewer for their positive and constructive feedback. We have now included several additional experiments based on the reviewer's suggestions that helped to further strengthen our work.

1. **Registry choice, potential biases:**

We appreciate the reviewer's insightful comments concerning the representativeness of our dataset and the recommendation to conduct additional experiments to identify potential model biases.

While we agree that including additional cohorts is generally beneficial and would be essential prior to clinical application, we currently lack access to further extensive CIJD databases. In this study, we focus on a retrospective analysis of the SCQM cohort, and we interpret our results in this scope (i.e. we contextualize our findings to this data). Notably, the SCQM registry, which captures the medical data of approximately 70% of Swiss rheumatologists, is highly representative of CIJD management in Switzerland. This database reflects real-world treatment across varied disease manifestations more accurately than typical clinical trial data.

Additionally, we are grateful for the reviewer's suggestion to explore spurious correlations within the model. As detailed in Appendix S1 Text, "Impact of Features on Prediction," particularly in Subsection 1.3 "Feature Associations Experiments," we conducted further analyses examining the features known to correlate with disease activity by perturbing their values and evaluating the impact on model predictions. Our findings indicate that while the model recognizes associations between certain features and increased disease activity, it does not exhibit systematic biases. Instead, it bases its predictions on the complex interplay of wide range of input features.

2. **Choice of DAS28/ASDAS scores:**

We appreciate the reviewer's feedback regarding the exclusion of patients lacking ASDAS/DAS28 scores. We acknowledge the importance of developing a model that is fair and works efficiently for as many patients as possible. For this analysis, we chose to focus on DAS28 and ASDAS scores as they are the most widely recognized measures of disease activity in rheumatoid arthritis and Axial Spondyloarthritis, respectively, which are areas of expertise for our collaborating rheumatologists. While, we agree that it would be interesting and beneficial to train the model for additional scores before deployment, excluding patients without any of these two scores only resulted in a data loss of about 2%. Moreover, the features of the excluded patients fall within distribution of our training data. We can thus safely hypothesize that the model should work effectively if tasked to predict DAS28/ASDAS values for these patients.

3. **Transparency of model predictions and similarity analysis:**

We thank the reviewer for pointing out concerns regarding the transparency of model predictions and patient similarities. We believe that the reviewer's concerns apply to any deep learning solution due to their inherent "black box" nature, and the inherent tradeoff between model's explainability and predictive power. Despite this trade-off, our model includes three layers of explainability: (a) attention weights, (b) feature importance from patient similarity, and (c) SHAP values, to provide insights into its functioning.

Inspired by the reviewer's suggestion, we have further enhanced our model's transparency by integrating an additional framework detailed in Section 3.2.4 and illustrated in Figures 11 and 12. The framework relates the model's prediction and similarity analysis (i.e. finding similar patient trajectories to the current index patient) to intuitive visual explanations. Clinicians could use this framework in practice to easily visualize which features impact model predictions the most at any time step. Furthermore, this use case includes an additional example patient for the similarity analysis, and additional example patients can be found in Appendices S6 Fig and S7 Fig. While it would indeed be beneficial to include genetic data in our similarity analysis to enrich our understanding, we currently do not have access to such data. Nonetheless, we are committed to improving the comprehensibility and application of our model within the limits of available resources.

4. **Model evaluation and robustness**

We fully agree with the reviewer that prospective testing is needed before deploying any model. In this study, we perform a retrospective analysis of CIJD patient management in Switzerland and the results should be interpreted within this context.

We thank the reviewer for suggesting to include monitoring results of patients across diverse subgroups to ensure fairness and robustness. We have expanded our analysis in Appendix S1 Text ("Error Analysis") to include the prediction error relative to feature values. For continuous features, we examined the correlation between feature values and model error, while for categorical features, we assessed the standard deviation of the prediction error across different categories, confirming that our model performs reasonably consistently across diverse patient subgroups.

Lastly, we now also included an additional experiment relating feature importance to prediction error in Appendix S1 Text, "Imputation Experiments", where we showed that imputing important features leads to higher prediction error and that imputing less important features has almost no impact on model predictions.

Universität Zürich[UZH]

Point by point response: Reviewer 2.

We thank the reviewer for their positive and constructive feedback. We have now included several additional experiments based on the reviewer's suggestions that helped to further strengthen our work.

1. **Dataset description**

Following the reviewer's guidance, we now included an extensive description of the feature distributions in figures S2 and S3 of the Appendix. As suggested, we specifically examined separately the features for patients who had fewer than three medical visits, showing that there are no significant population skews. In Figure S4 of the appendix, we furthermore plotted the mean and standard deviation of the DAS28/ASDAS scores for the different number of medical visits. Only the value at the first medical visit is on average slightly higher. It is important to note that our model does not predict values for the first medical visit.

We could not include patient ethnicity in our analysis as it is not recorded in the database. We have now acknowledged this limitation in the conclusion (lines 529-532).

Lastly, as suggested, we have now included a plot showing number of medical visits per patient in Figure S1 in the appendix.

2. **Data split**

We thank the reviewer for their question related to the strategy we used to perform the train-validation-test split of the data. We wish to clarify that we did not use a random data split. Instead, we employed a stratified split based on the number of medical visits, to ensure that the proportion of patients is similar for each number of available medical visits across all subsets of the data (cf line 214 in the manuscript and Figure 2 "project pipeline"). Moreover, the splitting is based on patients IDs such that patients assigned in the training set were not part of the test set and vice versa.

3. **Temporal baseline**

We thank the reviewer for suggesting to include a temporal ML baseline in addition to the static ML baselines. We now trained an additional LSTM baseline, using the patient histories as input. We describe this new baseline in lines 292-297 and compare the performance in Tables 1 and 2.

4. **Relevance for clinicians**

We thank the reviewer for their suggestion to emphasize the link between our model results and their clinical relevance. In the paper discussion, we included several paragraphs such as "Clinical relevance of findings" following each main analysis (pp12 and 15). These

paragraphs were mainly written by collaborating rheumatologists, relating the model findings to established medical knowledge. Moreover, to further explain the trajectories in figure 5, we now included an additional use case in section 3.2.4 and included Figures 11 and 12, to provide additional visual explanations about model predictions and showcasing how clinicians could use these explanations in practice.

5. **Choice of k for KNN**

We now included two plots in the Appendix Figure S5 justifying the choice of k = 50. It achieved the best performance on validation data. When too few or too many patients are used, the error increases.

6. **Data imputation analysis**

We thank the reviewer for suggesting this interesting experiment. In Appendix S1 Text “Imputation experiments”, we now perform the following experiment to analyze the impact of feature imputation on model predictions. For a given feature, we randomly imputed as missing 10% of the available values and computed the model error on this imputed dataset. We found that the impact on prediction error relates closely to the degree of importance of the feature, as determined by the patient similarity analysis.
